# Atrial Fibrillation as a Rare Complication of the Use of Nifedipine as a Tocolytic Agent: A Case Report and Review of the Literature

**DOI:** 10.1155/2018/8085649

**Published:** 2018-05-15

**Authors:** Nikolina P. Docheva, Emily D. Slutsky, Roger Sandelin, James W. Van Hook

**Affiliations:** Department of Obstetrics and Gynecology, University of Toledo, Toledo, OH, USA

## Abstract

Calcium channel blockers are commonly used tocolytic agents on Labor and Delivery units worldwide as part of the management of preterm labor. Despite their overall reassuring safety profile, rare cardiovascular complications have been reported. In this report, we describe the case of threatened preterm labor managed with nifedipine with subsequent development of atrial fibrillation. This type of cardiac arrhythmia may have considerable consequences for both the mother and the fetus. The aim of this case report and comprehensive review of the literature is to raise awareness.

## 1. Introduction

Normal maternal physiologic adaptations that occur during pregnancy can predispose to cardiac arrhythmias. Some of these adaptations are increased circulating blood volume and cardiac output with subsequent myocardial stretching, reduced systemic vascular resistance, modest decline in blood pressure, high plasma catecholamine concentrations, and adrenergic receptor sensitivity [1–5]. Studies have shown that the most common arrhythmias in pregnancy are ectopic premature contractions and nonsustained arrhythmias [6, 7]. Atrial fibrillation is rare in pregnancy with prevalence of 2 per 100,000 pregnancies and accounts for 1% of all hospital admissions for arrhythmia in the pregnant patient [7]. Most of the cases described in the literature occur in women with preexisting heart conditions such as congenital heart disease, hyperthyroidism, electrolyte disturbances, or the use of either recreational or prescribed drugs [8–10]. Atrial fibrillation, in the absence of structural heart disease or known cause, also known as “lone atrial fibrillation,” is even more rare [10–16]. We present a case report of a patient without any structural cardiac defects who developed atrial fibrillation following the administration of nifedipine as a tocolytic agent.

## 2. Case Presentation

A 25-year-old African American woman, Gravida 7, Para 1, Aborta 5, presented at 29 weeks and 2 days with threatened preterm labor. The patient initially sought care at an outside facility where she received 0.25 mg of terbutaline SC for tocolysis and 12 mg IM of betamethasone for lung maturation. The patient was transferred to our tertiary facility with strong, regular uterine contractions. She underwent a transvaginal ultrasonogram which showed a normal cervical length of 3.4 cm. The patient was placed on continuous cardiotocographic monitoring and started on nifedipine (Procardia) 20 mg every 4 hours, with subsequent administration of the second dose of 12 mg IM betamethasone. Her pregnancy was complicated by opioid abuse, normocytic anemia (hemoglobin on admission 9.9 g/dL), and history of low transverse cesarean section for breech presentation. During the course of her hospitalization, she complained of heart palpitations and chest pain that radiated to her neck. On examination, her pulse palpated as irregularly irregular and vitals revealed a tachycardia into the 140 s. A twelve-lead ECG confirmed atrial fibrillation with rapid ventricular response. Cardiology was consulted. The patient was transferred to the intensive care unit and began on diltiazem drip and intravenous metoprolol for rate control. She received a total of six doses of nifedipine during her admission before discontinuation of the medication. Her symptoms occurred within 20 hours from the first dose of nifedipine. Work-up included an echocardiogram, lower extremities venous Doppler, troponin levels, thyroid function test, electrolytes, liver function tests, and a repeat urine drug test. All results were normal apart from borderline magnesium of 1.7 mg/dL (see Table 1 for further results). The patient converted to normal sinus rhythm in less than 24 hours with a CHA_2_DS_2_-VASc score of 1 and anticoagulation with 81 mg aspirin was started. After transfer out of the intensive care unit the patient remained in sinus rhythm for the remainder of the hospitalization. Discharge medications included metoprolol 25 mg twice daily for rate control with close outpatient follow-up with MFM and cardiology.

The pregnancy culminated with a repeat low-transverse cesarean section at 39-week gestation resulting in a live-born male infant with Apgar scores of 9 and 9 at 1 and 5 minutes, respectively, and birthweight of 3400 grams. Continuous cardiac monitoring for 24 hours following delivery showed sinus rhythm. Per cardiology recommendations she was continued on metoprolol for prophylactic rate control and 81 mg aspirin in the postpartum period until she was seen as an outpatient. During that visit, metoprolol was discontinued.

## 3. Discussion

It is well known that atrial fibrillation predisposes to hemodynamic abnormalities and thromboembolic events leading to significant morbidity and mortality [17]. Pregnancy is a hypercoagulable state [18, 19] with an increased cardiac workload and therefore an increased susceptibility to arrhythmias [1–5]. Women with a history of any type of arrhythmia prior to pregnancy are at an increased risk of cardiac related morbidity, such as stroke and heart failure, during pregnancy [20]. However, because atrial fibrillation is rare in pregnancy, as are the sequela, it is difficult to adequately study the related adverse events. Lee et al. studied atrial fibrillation in pregnancy; of 264,730 pregnancies, atrial fibrillation was noted in only 157 [21]. The results suggest that older women (≥30 years of age) had higher odds of developing atrial fibrillation, and the odds significantly increased with increasing age [e.g., age 30–34 OR 4.1 95% CI (2.0–9.4), *p* < 0.001, and age ≥40 OR 5.2 95% CI (2.0–14.10), *p* < 0.001]. They also reported an increased prevalence among White and Black women as compared to Asian and Hispanic patients (111.6, 101.7, 45.0, and 34.3 per 100,000, resp.). Moreover, the odds for atrial fibrillation were higher during the third trimester compared to the first trimester of pregnancy [3.2 95% CI (1.5–7.7), *p* = 0.002]. In addition, there was no difference in birthweight among fetuses born to mothers with or without atrial fibrillation. However, the rate of admission to the neonatal intensive care unit was higher in the atrial fibrillation group (10.8% versus 5.1%  *p* = 0.003) [21].

The case described* here-in* of threatened preterm labor depicts a common scenario seen in Labor and Delivery units worldwide. Preterm birth affects 5 to 18% of pregnancies and is a leading cause of infant morbidity and mortality [22]. Inhibition of uterine contractility with tocolytic agents is central to the treatment for preterm labor. Many different agents are available to inhibit uterine contractions including calcium channel blockers, magnesium sulfate, nonsteroidal anti-inflammatory drugs, beta-adrenergic receptor agonists, and oxytocin antagonists. The choice of a tocolytic agent is largely based on its contraindications. A recent systematic review and meta-analysis of randomized controlled trials (*N* = 2,179 women) showed that nifedipine was associated with a significant reduction in the risk of preterm delivery within 7 days of starting treatment, when initiated before 34-week gestation, compared with beta-adrenergic receptor agonists. The analysis also showed that there was no difference between the tocolytic efficacy of nifedipine and magnesium sulfate. However, nifedipine had significantly less maternal adverse events than magnesium sulfate and beta-adrenergic receptor agonists [23].

Nifedipine is a dihydropyridine calcium channel blocker that causes smooth muscle relaxation with vasodilatory action in the peripheral vasculature. There is a potential to provoke a compensatory adrenergic drive in order to maintain cardiac output, resulting in reflex tachycardia [24]. As previously mentioned the maternal physiologic adaptations of pregnancy predispose to arrhythmias and the additional effects of nifedipine on the maternal cardiovascular system can increase the risk of developing an arrhythmia. Despite the relatively safe profile of nifedipine, case reports have been published in the literature which described patients developing myocardial infarction [25, 26], severe hypotension leading to fetal demise [27], maternal hypoxia [28], and pulmonary edema associated with nifedipine [29, 30]. Moreover, a study showed cases of nifedipine-associated maternal dyspnea in patients with twin pregnancies underscoring a concern for administering nifedipine in women with compromised cardiovascular conditions which could be due to multiple gestation, maternal hypertension, cardiac disease, or intrauterine infection [24]. Our patient received a total of 6 doses of nifedipine 20 mg and we believe that this predisposed her to develop paroxysmal atrial fibrillation. In addition, she was anemic, further increasing the strain on the myocardium and potentiating the effects of the nifedipine. Parasuraman et al. [31] published the first case report in which a patient with threatened preterm labor was treated with nifedipine and developed atrial fibrillation that responded to DC cardioversion, whereas Cheung et al. [32] described a case of maternal atrial fibrillation after sequential use of nifedipine and atosiban for the treatment of preterm labor. de Heus et al. reported, in a multicenter prospective cohort study, that, among the 542 women treated with nifedipine, 5 (0.9%) had a serious adverse side effect and 6 (1.1%) had a mild adverse side effect [33]. In addition, Khan et al., in a systematic review and meta-regression analysis, evaluated the safety of nifedipine as a tocolytic agent in preterm labor and as an antihypertensive agent in the treatment of hypertension in pregnancy. The results showed that adverse events were the highest among women given more than 60 mg total dose of nifedipine [OR 3.78, 95% CI (1.27–11.2), *p* = 0.017] and in reports from case series compared to controlled studies [OR 2.45, 95 CI (1.17–5.15), *p* = 0.018] [34]. Outside pregnancy, short acting nifedipine has been associated with increased risk of myocardial infarction and mortality when used to treat hypertension. This is believed to be due to the hypotension and reflex tachycardia that can predispose to an arrhythmia [35]. The FDA states that atrial or ventricular dysrhythmias can occur in less than 1% of patients on short acting nifedipine [36].

This case report provides a lesson for physicians in the field of high risk obstetrics. Caution must be taken when administering tocolytic agents such as nifedipine. Even though the medication is overall safe and the aforementioned side effects are rare, there is a small subset of patients that develop severe side effects such as atrial fibrillation, which may lead to significant maternal and fetal morbidity.

## Figures and Tables

**Table 1:** Laboratory results.

Laboratory test	Result	Normal range
Hemoglobin (g/dL)	10.4	11.7–15.5
Hematocrit (%)	30.8%	35–47%
CK-MB (ng/mL)	1.0	0.0–4.9
Total CK (U/L)	15	24–170
Ck relative index (Units)	6.7	0.0–2.4
Troponin I (ng/mL)	<0.01	0.00–0.04
Sodium (mmol/L)	134–146	142
Potassium (mmol/L)	4.2	3.5–5.0
D-dimer (ng/mL)	<255	340
AST (U/L)	17	0–41
ALT (U/L)	8	0–31
Magnesium (mg/dL)	1.7	1.8–2.6
TSH (uIU/mL)	1.95	0.49–4.67
T4 (ng/dL)	0.76	0.61–1.60
